# Dispensing by Nurses

**Published:** 1898-12-03

**Authors:** 


### Editor's Letter-Box.

**Our Correspondents are reminded that prolixity is a great bar to publication, and that brevity of style and conciseness of statement greatly facilitate early insertion.]**

### DISPENSING BY NURSES.

By the courtesy of Dr. Ackersley, we have been favoured with a full account of the circumstances attending the unfortunate case of over-dosing with tincture of opium at the Tolworth Isolation Hospital, on which we commented last week. He adds, "The verbal and written instructions were perfectly clear, and no one has at any time or in any way ventured for a moment to suggest the contrary. The matron and nurse were both represented by legal advisers at the inquest (I was not), and there was not the least attempt made to shift even a part of the blame on to me." We need hardly say that our remarks were directed against the system rather than the individual. We think that in the ordering of drugs it is a matter of some importance to consider by whom they will have to be dispensed, and that when the dispensing has to be done by other than trained pharmacists, every precaution should be taken to make the prescription as plain as possible. The illegibility of ordinary prescriptions is notorious, and when they are to be dispensed by nurses they should be written for nurses. In regard to the particular case in question, it is clear from Dr. Ackersley's account that the blame rests elsewhere, but that does not lessen the importance of the point which we raised.

